# A cross-sectional study of different patterns of oral contraceptive use among premenopausal women and circulating IGF-1: implications for disease risk

**DOI:** 10.1186/1472-6874-11-15

**Published:** 2011-05-20

**Authors:** Kristina M Blackmore, Jody Wong, Julia A Knight

**Affiliations:** 1 Prosserman Center for Health Research, Samuel Lunenfeld Research Institute, Mount Sinai Hospital, Toronto, Canada; 2 Dalla Lana School of Public Health, University of Toronto, Toronto, Canada

## Abstract

**Background:**

Insulin-like growth factor-1 (IGF-1) is important in normal growth, development, and homeostasis. Current use of oral contraceptives (OC) decreases IGF-1 concentrations; however, the effect of past use, age/timing of use, and type of OC used on IGF-1 levels is unknown. OC are the most commonly used form of birth control worldwide. Both IGF-1 and OC use have been linked to premenopausal breast and colorectal cancers, osteoporosis and cardiovascular disease (CVD). Understanding the effects of different patterns of OC use on IGF-1 levels may offer insight into its influence on disease risk in young women.

**Methods:**

In a cross-sectional study of 328 premenopausal women ages 18 to 21 and 31 to 40 we examined the relationship between different patterns of OC use and circulating IGF-1 using adjusted linear regression analysis. Information on OC use was obtained through an interviewer administered questionnaire. Plasma IGF-1 was assessed with enzyme linked immunosorbent assay (ELISA).

**Results:**

Among women aged 18 to 21, ever OC use was significantly associated with decreased IGF-1 levels compared to never use (β = -57.2 ng/ml, 95% confidence interval (CI): -88.7, -25.8). Among women aged 31 to 40, past users who first used OC at 25 years of age or older (β = 43.8 ng/ml, 95% CI: 8.8, 78.8), in the last 15 years (β = 35.1 ng/ml, 95% CI: 9.3, 61.0) or after 1995 (β = 46.6 ng/ml, 95% CI: 13.4, 79.8) had significantly higher IGF-1 levels compared to never users.

**Conclusion:**

This is the first study to highlight the long term effects of OC use after cessation on IGF-1 levels among premenopausal women, which previously were thought to be transitory. Future studies of past use and IGF-1 levels are required and must consider age/timing of use and type/generation of OC used. Additional studies are needed to confirm the potential mediation of IGF-1 levels in the links between OC use and health outcomes.

## Background

Insulin-like growth factor-I (IGF-I) is a growth hormone (GH) dependent peptide produced primarily in the liver, but it is also produced locally by many other tissues and can act in an autocrine and paracrine manner as well as through endocrine pathways [1]. IGF-1 plays an important role in physiological growth, development and homeostasis. Circulating IGF-1 concentrations have been used as an indicator of the total pool of IGF-1 available to cells and as a reflection of inter-individual differences in IGF-1 tissue bioactivity [2].

The relationship between circulating IGF-1 levels and disease risk among women is complex as studies have shown that IGF-1 can be either positively (e.g. cancer) or negatively (i.e. osteoporosis, heart disease) associated with adverse health outcomes [3-11]. In both normal and neoplastic cells IGF-1 regulates cell proliferation, mitogenesis and apoptosis [12] and high IGF-1 levels have been associated with approximately twice the risk of developing premenopausal breast cancer and with a non-significant two fold increased risk of colorectal cancer [3-7]. IGF-I is also critical for long bone growth and skeletal maturation and in adulthood it helps to maintain bone mass and density [13]. Among postmenopausal women, high IGF-1 levels are associated with a reduced risk of osteoporotic fractures; a positive correlation between serum IGF-1 levels and bone mineral density is also evident [8,9]. In the heart, IGF-1 delays cardiomyocyte apoptosis, affects myocardial contractility, and participates in the inflammation linked angiogenesis and repair processes following ischaemic events [14]. Consequently, high IGF-1 levels have been associated with a reduced risk of both ischemic heart disease (IHD) and ischemic stroke (IS) [10,11,14].

Because of the role IGF-1 plays in health and disease, there has been a growing interest in understanding factors that influence IGF-1 levels [15-17]. Age is a strong predictor of circulating IGF-1; ethnicity, anthropometric indices (body mass index (BMI), weight, height), physical activity, smoking, alcohol and diet can also affect IGF-1 levels [15,17-22]. A small number of studies have examined the effect of current use of oral contraceptives (OC) on IGF-1 concentrations; when compared to never users current users have significantly lower IGF-1 levels [23-25]. However, the effect of other patterns of OC use, in particular past use and timing of use during life on circulating IGF-1 has not been studied. These patterns may influence IGF-1 levels over the longer term and thus also impact IGF-1 related health outcomes. In addition, many earlier studies had limitations such as small study samples and failure to control for factors known to influence circulating IGF-1, such as age, BMI and ethnicity [23-25], thereby limiting current knowledge of the relationship between OC use and IGF-1 levels.

We examined the association between other patterns of use not measured in earlier studies, such as past use, and plasma IGF-1 levels among 328 premenopausal women aged 18 to 21 and 31 to 40. The association between the generation of OC (i.e. 2^nd ^versus 3^rd^) women likely used and IGF-1 was also explored. Numerous studies have shown an association between OC use and risk of breast and colorectal cancers, osteoporosis and cardiovascular disease (CVD) [26-37]. IGF-1 is a key player in health and disease and a few studies have suggested a relationship between OC use and IGF-1 levels. Hence, understanding how OC use affects circulating IGF-1 may provide insight into whether the relationship between OC use and disease risk may in part be mediated by the IGF-1 pathway.

## Methods

### Recruitment of study population and data collection

The women in this analysis were initially recruited for a previously completed study [38]. In the earlier study a total of 368 women were recruited in Toronto, Canada from July 2005 to July 2008 through advertising at local universities, hospitals, community centres and newspapers. Women were recruited into two groups: those aged 18 to 21 who were nulliparous (n = 200) and those aged 31 to 40 who were either nulliparous or parous (n = 168). Of the 368 women recruited, 40 women were excluded because of 1) missing IGF-1 values (n = 4); 2) missing information on the date of their last menstrual period (n = 9); 3) not having had a menstrual period within 42 days among 18 to 21 year olds (n = 5) and within 35 days among 31 to 40 year olds (n = 1) [39]; 4) missing information regarding OC use (n = 21). This left a total of 328 women for the present analysis: 180 women aged 18 to 21 years and 148 women aged 31 to 40 years. At a single study visit for each woman we measured her height, weight and a blood sample was drawn. Information on OC use and other variables was obtained through an interviewer administered questionnaire. Women were asked if they had ever used OC, the age at which they initiated and/or discontinued use (where applicable), and if they were currently using OC. All procedures were reviewed and approved by the Research Ethics Boards of Mount Sinai Hospital and the University Health Network and participating women provided informed consent.

### IGF-1 assay

For each woman an aliquot of plasma was thawed and IGF-1 was assessed with enzyme linked immunosorbent assay (ELISA) using the DSL total IGF-1 kit (DSL-10-2800). Intra-assay coefficients of variation were 5% to 9%; inter-assay coefficients of variation were 9% to 11%.

### Data Analysis

The effect of OC use (ever vs. never) on IGF-1 levels differed according to age group (18 to 21 years vs. 31 to 40 years) (*P *for interaction = 0.005). Consequently, we assessed the relationship between different patterns of OC use and IGF-1 levels separately in women aged 18 to 21 and women aged 31 to 40 using linear regression. The patterns of OC use considered in each age group were: ever vs. never use; current vs. past vs. never use; age (years) at first use (for ages 18 to 21: 10 to 15; 16 to 18; 19 to 21; for ages 31 to 40: <= 18; 19 to 24; 25+); duration (years) of use (for ages 18 to 21: < 1; 1 to 2; > 2; for ages 31 to 40: < 5; 5 to 9; 10+); and time (years) since last use (for ages 18 to 21: Current; < 1; 1+; for ages 31 to 40: Current, < 5; 5 to 9; 10+). Among women aged 31 to 40 we also considered time (years) since first use (< 15; 15 to 19; 20+) and year of first use (1979 to 1994; 1995-2004). Women who reported ever using OC (at least one month of use) were considered *ever *users. Among *ever *users, women using OC at the time of their study visit were considered *current *users; women who stopped using OC prior to their study visit were considered *past *users. For analyses including women ages 18 to 21 years, the following confounders were included in the adjusted models as independent variables: age (age 18 vs. older) and ethnicity (East Asian, South Asian, Other/Mixed vs. European). Among women 31 to 40 years old, adjusted models included the following confounders as independent variables: weight (75^th ^percentile and above vs. below), current physical activity (ever doing an activity at least once per week vs. never) and the number of days since their last menstrual period (<= 14 vs. > 14). These confounders were selected as they showed some association with IGF-1 levels in unadjusted analyses. All tests were two-sided with *P *< 0.05 as the criterion for significance. All analyses were conducted using SAS version 9.2.

## Results

Table 1 displays the characteristics of the study population. Mean IGF-1 levels were significantly higher among younger women compared to older women (*P *< 0.0001). The majority of women aged 18 to 21 were either East Asian or Caucasian, while the majority of women aged 31 to 40 were Caucasian. Younger women were more likely to report never use of OC, whereas older women were more likely to report past use.

**Table 1:** Characteristics of the study population, women aged 18 to 21 years and aged 31 to 40 years.

	Ages 18 to 21 (n = 180)		Ages 31 to 40 (n = 148)	
	Mean (SD)	Range	Mean (SD)	Range
IGF-1 (ng/ml)	315.4 (104.1)	94.0 - 620.0	174.4 (56.7)	33.0 - 314.0
Age (years)	19.7 (1.1)	18 - 21	35.3 (3.1)	31 - 40
BMI (kg/m^2^)	23.3 (4.2)	17.5 - 48.9	25.6 (5.5)	17.4 - 50.8
Height (cm)	162.8 (6.9)	146.0 - 183.0	162.7 (5.7)	145.0 - 175.0
Weight (kg)	62.0 (13.7)	44.6 - 139.6	67.9 (15.0)	44.0 - 135.1
Days since last period	16.7 (9.7)	1.0 - 41.0	15.9 (8.9)	1.0 - 35.0
Ethnicity n(%)				
*European*	69 (39)		97 (65)	
*East Asian*	56 (31)		11 (7)	
*South Asian*	17 (10)		7 (5)	
*Other/Mixed*^*a*^	37 (21)		34 (23)	
Parous n(%)	0 (0)		36 (24)	
OC use n(%)				
*Never*	100 (56)		37 (25)	
*Past*	29 (16)		88 (59)	
*Current*	51 (28)		24 (16)	
Ever physical activity n(%)^b^				
*Childhood*	169 (94)		127 (85)	
*Teenage*	149 (83)		118 (79)	
*Current*	129 (72)		126 (85)	

^a ^Includes African, Caribbean, Middle Eastern, First Nation, Latin American; ^b ^Doing physical activity at least once per week

Table 2 shows unadjusted IGF-1 levels according to different characteristics for women aged 18 to 21 and women aged 31 to 40. Among women aged 18 to 21, IGF-1 levels were significantly higher in women aged 18 years old compared to those aged 19 to 21 years old. IGF-1 levels were lowest among East Asians and women of Other/Mixed ancestry compared to Europeans and South Asians and the difference across groups approached significance. Among women aged 31 to 40 years, women who were in the highest quartile of weight had lower IGF-1 levels compared to those in the lower three quartiles and the difference across groups was significant. Women who reported currently doing any physical activity at least once per week had significantly higher IGF-1 levels compared to those who did not. IGF-1 levels were also significantly higher in the luteal phase of the menstrual cycle (> 14 days since the last menstrual period) compared to the follicular phase.

**Table 2:** IGF-1 levels (ng/ml) for women aged 18 to 21 years and women aged 31 to 40 years according to different characteristics.

	Ages 18 to 21 (n = 180)				Ages 31 to 40 (n = 148)		
	**No**.	Mean (SD)	P*		**No**.	Mean (SD)	P*
Age			*0.002*				*0.76*
*18*	32	366.9 (121.5)		*31 to 35*	81	175.7 (61.0)	
*19 to 21*	148	304.3 (96.9)		*36 to 40*	67	172.8 (51.4)	
BMI (kg/m^2^)			*0.36*				*0.12*
*< 20.0*	33	312.1 (93.8)		*< 25.0*	82	178.8 (55.2)	
*20.0 - 24.9*	107	323.6 (109.3)		*25.0 - 29.9*	41	178.8 (62.7)	
≥ *25.0*	40	296.3 (97.4)		≥ *30.0*	25	152.9 (47.9)	
Height (cm)			*0.67*				*0.92*
≤ *158.5*	52	311.3 (109.8)		≤ *159.5*	39	176.2 (61.8)	
*158.6 - 162.4*	42	322.6 (101.2)		*159.6 - 163.0*	46	174.6 (56.8)	
*162.5 - 167.4*	43	327.2 (102.6)		*163.1 - 167.0*	35	168.9 (46.7)	
≥ *167.5*	43	301.7 (103.1)		≥ *167.1*	28	178.5 (63.0)	
Weight (kg)			*0.14*				*0.02*
≤ *53.5*	44	316.3 (101.6)		≤ *57.9*	35	170.4 (48.8)	
*53.6 - 59.3*	47	301.1 (95.6)		*58.0 - 64.4*	37	186.7 (62.0)	
*59.4 - 66.3*	45	344.6 (122.2)		*64.5 - 73.4*	40	186.7 (52.5)	
≥ *66.4*	44	300.0 (91.6)		≥ *73.5*	36	152.1 (57.6)	
Ethnicity			*0.08*				*0.90*
*European*	69	327.2 (112.8)		*European*	96	172.7 (54.6)	
*East Asian*	56	304.3 (108.6)		*East Asian*	11	186.1 (84.7)	
*South Asian*	17	357.9 (92.9)		*South Asian*	7	171.4 (50.0)	
*Other/Mixed*^*a*^	37	288.8 (76.4)		*Other/Mixed*^*a*^	34	176.2 (55.3)	
Parity							*0.49*
*Nulliparous*	180	-		*Nulliparous*	112	176.3 (57.5)	
*Parous*	0	-		*Parous*	36	168.7 (54.7)	
Physical Activity^b^							
Childhood			*0.29*				*0.17*
*Never*	11	283.3 (81.5)		*Never*	22	159.1 (64.0)	
*Ever*	169	317.5 (105.3)		*Ever*	126	177.1 (55.2)	
Teenager			*0.68*				*0.98*
*Never*	31	322.4 (103.1)		*Never*	31	174.2 (60.6)	
*Ever*	149	314.0 (104.6)		*Ever*	117	174.5 (55.9)	
Current			*0.78*				*0.04*
*Never*	50	319.2 (116.6)		*Never*	23	151.9 (60.8)	
*Ever*	129	314.3 (99.7)		*Ever*	125	178.6 (55.2)	
Days since last period			*0.77*				*0.02*
≤ *14*	72	312.6 (100.9)		≤ *14*	66	162.1 (50.7)	
*>14*	108	317.3 (106.7)		*> 14*	82	184.4 (59.6)	

* Results of t-test, one-way ANOVA (where applicable); ^a ^Includes African, Caribbean, Middle Eastern, First Nation, Latin American; ^b ^Doing physical activity at least once per week

The results of linear regression analysis for the relationship between different patterns of OC use and IGF-1 levels for women aged 18 to 21 and women aged 31 to 40 are shown in Tables 3 and 4, respectively. Among women 18 to 21 years of age, those who ever used OC, either past or current users, had significantly lower IGF-1 levels compared to those who never used them. Among those who ever used OC, we observed no relationship between age at first use, time since last use or total duration of use and IGF-1 levels. In the adjusted model, ever use of OC explained approximately 9% (adjusted R-square (R^2^) = 0.09) of the total variation in IGF-1 levels in this age group.

**Table 3:** Results of linear regression analysis (unadjusted and adjusted) for the relationship between patterns of oral contraceptive use and IGF-1 levels (ng/ml) for women aged 18 to 21 years.

			Unadjusted			**Adjusted**^**a**^		
	**No**.	Mean (SD)	Beta	95%CI	*P*	Beta	95%CI	*P*
**OC Use**								
*Never*	100	336.8 (111.5)	Reference		*0.002*	Reference		*0.0004*
*Ever*	80	288.7 (87.6)	-48.1	-78.2, -18.0		-57.2	-88.7, -25.8	
								
*Never*	100	336.8 (111.5)	Reference		*0.007*	Reference		*0.002*
*Past*	29	295.6 (82.8)	-41.2	-83.6, 1.2		-51.7	-94.6, -8.8	
*Current*	51	284.7 (90.8)	-52.1	-86.6, -17.5		-60.2	-95.5, -24.9	
								
**Ever Users**								
								
**Age at first use**								
*10 to 15 years*	11	316.6 (103.4)	43.1	-23.2, 109.3	*0.44*	34.2	-36.7, 105.0	*0.61*
*16 to 18 years*	50	288.3 (87.9)	14.7	-32.4, 61.8		17.4	-44.7, 54.1	
*19 to 21 years*	19	273.6 (77.2)	Reference			Reference		
								
**Time since last use**								
*Current*	51	284.7 (90.8)	Reference		*0.67*	Reference		*0.60*
*< 1 year*	11	310.6 (29.3)	25.9	-32.5, 84.3		28.7	-30.0, 87.4	
≥ *1 year*	18	286.4 (102.7)	1.7	-46.5, 49.9		-1.7	-50.8, 47.5	
								
**Total duration of use**								
*< 1 year*	12	273.6 (78.8)	-14.8	-76.3, 46.6	*0.80*	-11.8	-77.3, 53.7	*0.82*
*1 to 2 years*	42	293.2 (86.7)	4.7	-39.2, 48.7		7.0	-37.6, 51.6	
*>2 years*	26	288.4 (95.1)	Reference			Reference		

^a ^Adjusted for age (18 vs. 19 to 21) and ethnicity (East Asian, South Asian, Other/Mixed vs. European)

**Table 4:** Results of linear regression analysis (unadjusted and adjusted) for the relationship between patterns of oral contraceptive use and IGF-1 levels (ng/ml) for women aged 31 to 40 years.

			Unadjusted			**Adjusted**^**a**^		
	**No**.	Mean (SD)	Beta	95%CI	*P*	Beta	95%CI	*P*
**OC Use**								
*Never*	37	167.2 (58.7)	Reference		*0.37*	Reference		*0.30*
*Ever*	111	176.8 (56.1)	9.6	-11.7, 30.9		10.8	-9.7, 31.3	
								
*Never*	37	167.2 (58.7)	Reference		*0.18*	Reference		*0.09*
*Past*	87	181.4 (56.9)	14.2	-7.7, 36.1		16.4	-4.7, 37.6	
*Current*	24	160.2 (51.1)	-7.0	-36.3, 22.2		-7.7	-35.6, 20.2	
**Age at first use**								
Never	37	167.2 (58.7)	Reference		*0.04*	Reference		*0.04*
Current	24	160.2 (51.1)	-7.0	-35.8, 21.7		-7.8	-35.4, 19.8	
Past								
≤ *18 years*	47	168.8 (52.7)	1.4	-22.5, 25.7		5.5	-17.8, 28.9	
*19 to 24 years*	28	188.8 (61.0)	21.5	-5.9, 49.0		22.5	-4.2, 49.1	
≥ *25 years*	12	213.8 (50.6)	46.5	10.1, 83.0		43.8	8.8, 78.8	
**Time since first use**								
Never	37	167.2 (58.7)	Reference		*0.01*	Reference		*0.03*
Current	24	160.2 (51.1)	-7.0	-35.4, 21.3		-7.5	-35.0, 19.9	
Past								
≥ *20 years*	25	156.0 (47.4)	-11.3	-39.3, 16.8		-1.4	-29.3, 26.4	
*15 to 19 years*	32	179.2 (54.5)	12.0	-14.2, 38.1		11.8	-13.7, 37.4	
*< 15 years*	30	205.0 (58.4)	37.8	11.2, 64.4		35.1	9.3, 61.0	
**Time since last use**								
Never	37	167.2 (58.7)	Reference		*0.35*	Reference		*0.30*
Current	24	160.2 (51.1)	-7.0	-36.4, 22.3		-7.7	-35.8, 20.4	
Past								
*<5 years*	26	183.0 (58.1)	15.7	-12.9, 44.4		14.4	-13.0, 42.0	
*5 to <10 years*	29	188.3 (61.9)	21.1	-6.7, 48.8		20.7	-6.1, 47.6	
≥ *10 years*	32	173.9 (51.7)	6.7	-20.3, 33.8		14.2	-12.0, 40.5	
**Duration of use**								
Never	37	167.2 (58.7)	Reference		*0.23*	Reference		*0.22*
Current	24	160.2 (51.1)	-7.0	-36.3, 22.2		-7.7	-35.7, 20.3	
Past								
*<5 years*	21	197.0 (65.2)	29.8	-0.7, 60.2		25.8	-3.6, 55.2	
*5 to < 10 years*	33	178.6 (50.2)	11.4	-15.3, 38.1		16.3	-9.5, 42.0	
≥ *10 years*	33	174.3 (57.4)	7.1	-19.6, 33.8		10.2	-15.7, 36.2	
**Year of first use**								
Never	37	167.2 (58.7)	Reference		*0.03*	Reference		*0.02*
Current	24	160.2 (51.1)	-7.0	-35.9, 21.8		-8.1	-35.6, 19.4	
Past								
*1979 to 1994*	73	175.4 (54.8)	8.2	-14.0, 30.4		10.8	-10.6, 32.2	
*1995 to 2004*	14	212.9 (59.2)	45.6	11.1, 80.1		46.6	13.4, 79.8	

^a ^Adjusted for current weight (75^th ^percentile and above vs. below), days since last reported menstrual cycle (<=14 vs. >14), and current physical activity at least once per week (ever vs. never)

Among women aged 31 to 40, current and never users had similar IGF-1 levels. Past users who first reported using OC at the age of 25 years or older and/or more recently (in the last 15 years or starting in 1995 or later) had the highest IGF-1 levels compared to never users. There was no association between IGF-1 levels and duration of use or time since last use in past users. In adjusted analyses comparing never vs. past vs. current users, OC use explained approximately 4% (adjusted R^2 ^= 0.04) of the total variation in IGF-1 levels in this age group, with a maximum adjusted R^2 ^of 0.09 when considering time since 1^st ^use in past users compared to never users. We observed no relationship between IGF-1 levels and different patterns of OC use among current users when compared to never users (data not shown).

**Figure 1:**
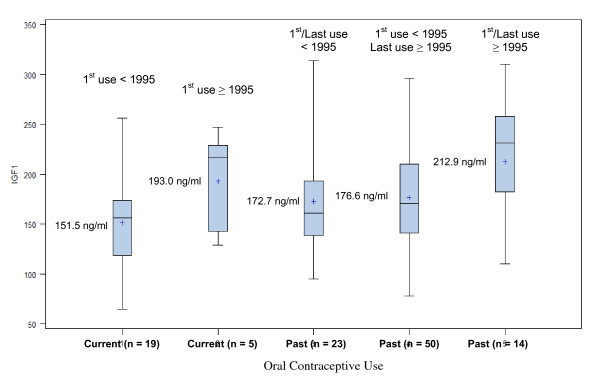
**Unadjusted mean IGF-1 levels (ng/ml) according to year of first use among past and current users of oral contraceptives aged 31 to 40 years**.

Figure 1 displays unadjusted IGF-1 levels according to year of first and last use among past users and according to year of first use among current users. We used year as a proxy for the type of OC women may have used. Among past users, those who first used OC from 1995 onward had significantly higher IGF-1 levels compared to both groups of women who first used OC prior to 1995 (*P *= 0.02). A similar pattern was observed among current users, but the difference in IGF-1 levels was not significant (*P *= 0.11). IGF-1 levels of ever users (past or current) who only used OC 1995 or later were also significantly higher compared to ever users who used OC before 1995 at some point (207.7 ng/ml vs. 170.5 ng/ml, *P *= 0.008).

## Discussion

The relationship between OC use and plasma IGF-1 levels differed between women ages 18 to 21 and women ages 31 to 40. Women in the younger age group who reported ever using OC had significantly lower IGF-1 levels compared to never users. Among older women, past users of OC had significantly higher IGF-1 levels compared to never and current users. Past users ages 31 to 40 who first used OC at 25 years of age or older and/or more recently (15 years ago or less; from 1995 onward) had the highest IGF-1 levels. Among older women the type of OC they may have used (i.e. 2^nd ^vs. 3^rd ^generation) influenced IGF-1 levels differently, with 3^rd ^generation OCs (introduced around 1995) [40] resulting in higher IGF-1 levels among both past and current users.

Previous studies of OC use and IGF-1 levels found lower circulating IGF-1 in current users, consistent with our findings among women ages 18 to 21 years. Among 311 women ages 17 to 35, current users had significantly lower plasma IGF-1 levels compared to non-users [23]. Similarly, in two smaller studies, women currently taking OC (n = 9, mean age 25.8 years) had significantly lower plasma IGF-1 levels compared to women receiving no medication (n = 10, mean age 31.0 years) [24] and 18 women compared before and after intake of either of two OC formulations over 21 days had significant reductions in IGF-1 levels [25].

Conversely, among women ages 31 to 40 we found that those who first used OC in adulthood and/or more recently and then stopped had increased IGF-1 levels compared to never and current users. To our knowledge, our study is the first to consider past use of OC and other patterns of OC use and IGF-1 levels in premenopausal women and suggests a potential long term effect of past use of OC on IGF-1 depending on when they are first started. The mechanism by which OC suppress circulating IGF-1 in current users is likely a pharmacological effect resulting from hepatic exposure to the estrogen component delivered via the portal circulation [24]. These effects are believed to be transitory and no long-term effects on the IGF-1 axis have been reported to date [15], but no longitudinal studies have examined the relationship between past use of OC and IGF-1. Also women who started taking OC at an older age and/or more recently may have done so for different reasons than women taking them at a younger age and this may in part explain the effect of past use at an older age on IGF-1 levels in this age group.

The effect of past use of OC on IGF-1 levels in older women may also be influenced by the type (generation) of OC they were using. Only two studies have considered the effect of different OC formulations on IGF-1 levels [23,25]. One study observed an inverse relationship between the average daily estrogen dose and IGF-1 levels, although no association was found between the type of progesterone and IGF-1 levels [23]. Conversely, in a short-term study in a small number of women, a greater reduction in mean plasma IGF-1 concentrations was found in women taking an OC formulation containing a third generation versus second generation progesterone [25]. In the present study, past users starting at a younger age most likely would have used second generation OC (introduced in the 1970s), at least initially, while those starting at an older age most likely would have used third generation OC (available circa 1995) [40]. Second and third generation OC contain a lower estrogen dose compared to earlier formulations, but are different from each other in the type of progesterone they contain (androgenic vs. non-androgenic progestogen, respectively) [25]. Contrary to earlier findings [25] we found that both past and ever users who likely used second generation OC (< 1995) had lower IGF-1 levels compared to past and ever users who likely used third generation OC (1995 or later). The effects of OC on circulating IGF-1 could be due to a number of factors in combination, including age at use, OC formulation, and current vs. past use.

Oral contraceptives are the most commonly used form of birth control by women worldwide [40]. However, their use has been linked to premenopausal breast and colorectal cancers, osteoporosis and CVD [26-37]. Current and recent use (1 to 9 years since last use) and an earlier age at first use (< 20 years) have been associated with an increased risk of breast cancer [26-28], while ever use of OC is associated with a reduced risk of colorectal cancer [29,30]. Studies in adolescent girls and premenopausal women suggest that current OC use has detrimental effects on bone mineral density, while studies of past use in peri/post-menopausal women suggest a benefit of OC on bone health, although some studies show no effect [31-33]. An increased risk of CVD has been reported with current OC use compared to non-current and never use; a small number of studies have found a decreased risk or no risk with past use when compared to never use [34-37].

Based on the findings of the present study and earlier ones, could the relationship between OC use and disease risk be at least in part mediated by the IGF-1 pathway? IGF-1 is positively associated with breast and colorectal cancers in some studies [3-7]. With respect to the positive association between OC use and breast cancer [26-28], if mediated through IGF-1, we would expect IGF-1 levels to be higher in current or recent users and/or women who started OC at a younger age. However, our results do not support this hypothesis. It is also unlikely that IGF-1 plays a role in the link between OC use and colorectal cancer risk as we would anticipate lower IGF-1 levels in past users who have a lower risk of colorectal cancer [29,30]. It should be noted that the relationship between 3^rd ^generation OC and breast and colorectal cancer has not yet been evaluated and may differ from relationships with older formulations. The relationship between OC use and CVD may, however, be mediated by IGF-1. Increased risk of CVD is associated with both current OC use and lower IGF-1 levels [10,11,34-37] and our results confirm reduced IGF-1 levels in current OC users. Decreased CVD risk with past OC use [34-37] may be due to higher IGF-1 levels in former users as we observed. Lower IGF-1 levels among current users may also potentially lead to decreased bone mineral density, particularly in young women, while the higher levels we observed in older past users may decrease osteoporosis risk, reflecting observed relationships between IGF-1, bone density and osteoporosis [8,9,13].

The limitations of our study include the lack of detailed information on the formulation of OC used, which may have contributed to the low R^2^, the reasons for starting and stopping OC, and missing information on some potential confounders including smoking and alcohol use. Strengths include the larger number of women compared to most previous studies and the availability of detailed information on the timing of initiation and cessation of OC. Few studies have addressed the potential effects of past use of OC or the type/generation of OC used.

## Conclusions

Our study is the first to demonstrate the long term effects of OC after cessation on IGF-1 levels, particularly in premenopausal women. It also highlights the importance of considering the timing of OC use during life and the type/generation of OC used in future studies of the health effects of OC use. Additional studies are needed to confirm the potential mediation of IGF-1 levels in the links between OC use and adverse health outcomes, such as CVD and osteoporosis.

## List of Abbreviations

BMI: body mass index; CI: confidence interval; CVD: cardiovascular disease; ELISA: enzyme linked immunosorbent assay; GH: growth hormone; IGF-1: Insulin-like growth factor-1; IHD: ischemic heart disease; IS: ischemic stroke; OC: oral contraceptives; R^2^: R-square;

## Competing interests

The authors declare that they have no competing interests.

## Authors' contributions

KMB was responsible for the initial concept, all data analysis, and for writing the submitted manuscript. JW was responsible for acquiring the data used in this analysis. JAK designed, directed and supervised all aspects of the initial study from which the data used in the present analysis was obtained and contributed to the current analysis and drafting of the manuscript. All authors reviewed and approved the final manuscript.

## Pre-publication history

The pre-publication history for this paper can be accessed here:

http://www.biomedcentral.com/1472-6874/11/15/prepub
